# Mechanism of TCONS_00147848 regulating apoptosis of nasal mucosa cells and alleviating allergic rhinitis through FOSL2-mediated JAK/STAT3 signaling pathway

**DOI:** 10.1038/s41598-021-94215-3

**Published:** 2021-08-06

**Authors:** Haiyun Huang, Yu Ren, Hongyu Liang, Xiaojia Liu, Jisangmo Nan, Hui Zhao, Xiaoling Liu

**Affiliations:** 1 ENT Department, Inner Mongolia People’s Hospital, Hohhot Inner Mongolia Autonomous Region, Hohhot, 010017 China; 2 Scientific Research Department, Inner Mongolia People’s Hospital, Hohhot Inner Mongolia Autonomous Region, Hohhot, 010017 China; 3 Medical Department, Inner Mongolia People’s Hospital, Hohhot Inner Mongolia Autonomous Region, Hohhot, 010017 China

**Keywords:** Cell biology, Molecular biology

## Abstract

This study was conducted to explore the roles and related mechanisms of lncRNA-TCONS_00147848 (TCONS_00147848) in nasal mucosa cell apoptosis and allergic rhinitis (AR). AR mice were sensitized with ovalbumin (OVA), with the TCONS_00147848 interference lentiviral vector (TCONS_00147848 shRNA) and FOSL2 overexpressing lentiviral vectors (pCDH-FOSL2) constructed respectively. NC shRNA, TCONS_00147848 shRNA and TCONS_00147848 shRNA + pCDH-FOSL2 were transfected into AR mice and mice with TNF-α induced nasal mucosa cells. The allergic reaction symptoms were evaluated by scoring. And in this study, we used Hematoxylin–Eosin (HE) staining and Terminal deoxynucleotidyl transferase dUTP nick end labeling (TUNEL) to detect the histological changes of nasal mucosa and apoptosis of nasal mucosa epithelial cells in mice, cell counting kit-8 (CCK-8) assay, Transwell and annexin V/PI to detect proliferation, migration and apoptosis of nasal mucosa cells of mice, respectively, enzyme-linked immunosorbent assay (ELISA) to detect the expression of inflammatory factors, qRT-PCR to detect TCONS_00147848 expression, Western blot assay to detect the expressions of FOSL2, JAK-2, STAT3, p-STAT3, BAX and BCL-2, RNA-binding protein immunoprecipitation (RIP) assay, RNA pull down assay and Co-immunoprecipitation (CoIP) assay to identify TCONS_00147848 targeting FOSL2. All these findings above reveal that knocking down TCONS_00147848 can reduce the allergic reaction symptom score of AR mice and the inflammatory reaction. The expression of IgE, IL-4, IL-5, IL-10, IL-9, IFN-γ and TNF-α in serum decreased. The expression of FOSL2, JAK-2, p-STAT3 and BAX in nasal mucosa and nasal mucosa cells of mice decreased as well, but BCL-2 expression increased. In addition, koncking down TCONS_00147848 can also inhibit the apoptosis of TNF-α induced nasal mucosa cells in mice and promote cell proliferation and migration. However, FOSL2 overexpression neutralized the effect of TCONS_00147848 shRNA. In nasal mucosa cells of mice, TCONS_00147848 can target FOSL2, interacting with STAT3. Inhibition of TCONS_00147848 can regulate JAK/STAT3 signaling pathway and reduce inflammatory response in AR mice.

## Introduction

Allergic rhinitis (AR) is a common and frequently occurring disease in clinical otorhinolaryngology, which is a chronic inflammatory reaction of nasal mucosa released by IgE mediated mediators after individual contact with allergens, and involves a variety of immune active cells and cytokines^1,2^. Its main clinical manifestations are nasal congestion, nasal itching, sneezing, runny nose, etc., showing seasonal or perennial attacks, with a certain impact on patients' daily life and social activities^3^.

lncRNA is a kind of non-coding RNA with a length of more than 200 nt, which can not encode proteins. The number of lncRNAs is much lower than that of coding genes, but its expression is specific in tissues and organs. Recently, researchers have found that lncRNAs play an important role in various human immune diseases^4,5^. However, their effects on AR pathology have not been fully understood. According to the sequencing results of our previously unpublished lncRNA, it was found that TCON_00147848 was highly expressed in the nasal mucosa of AR mice. The sequencing results were deposited at the National Center for Biotechnology Information (NCBI) (BioProject ID: PRJNA737061). Online database catRAPID (http://service.tartaglialab.com/page/catrapid_group) was used to predict, showing that there were possible regulatory targets between TCONS_0014784 and FOSL2.

FOSL2 participates has been found in immune regulation, which play an important role in chronic inflammatory diseases. Its overexpression can promote inflammatory response and inhibit T cell proliferation. In rats with sepsis, FOSL2 can promote hepatocyte apoptosis by activating JAK/STAT3 signaling pathway^6^. However, miR-30e and miR-133a can negatively regulate FOSL2 expression and reduce the damage of hepatocytes^7^. According to Lian et al., the expression of AFAP1-AS1/miR-423-5p mediated FOSL2 could stimulate Rho/Rac pathway and promote the migration of nasopharyngeal carcinoma cells^8^. And in a genetic study of allergic diseases by Ferreira et al., 11 previously unreported risk loci for allergic diseases were identified, including FOSL2^9^. Cheng et al. proved that the single nucleotide polymorphism of FOSL2 was associated with AR^10^. Based on previous studies, we speculated that TCONS_00147848 can target the FOSL2 mediated inflammatory response and affect the allergic symptoms of AR mice. In this study, we mainly studied the mechanism of TCONS_00147848 in AR.

## Methods

### TCON_00147848 shRNA and FOSL2 overexpression lentiviral vectors constructed respectively

TCON_00147848 shRNA and pcDH-FOSL2 were synthesized by upstream and downstream specific amplification primers, and restriction restriction sites were introduced, respectively, which were loaded into lentiviral plasmid vectors. Lentivirus shuttle plasmids and their auxiliary packaging original plasmids were prepared, and the high purity and endotoxin-free plasmids were extracted and co-transfected into HEK293T cells. Meanwhile, the lentiviral empty vector was transfected as the negative control. After transfection for 6 h, the complete medium was replaced. After culture for 48 h, cell supernatant rich in lentivirus particles were collected, respectively, and the virus supernatant was concentrated by super centrifugation. The virus titer was determined by fluorescence counting method, with the packaged virus used in the following experiment.

### Establishment of mice model of allergic rhinitis

**Figure 1:**
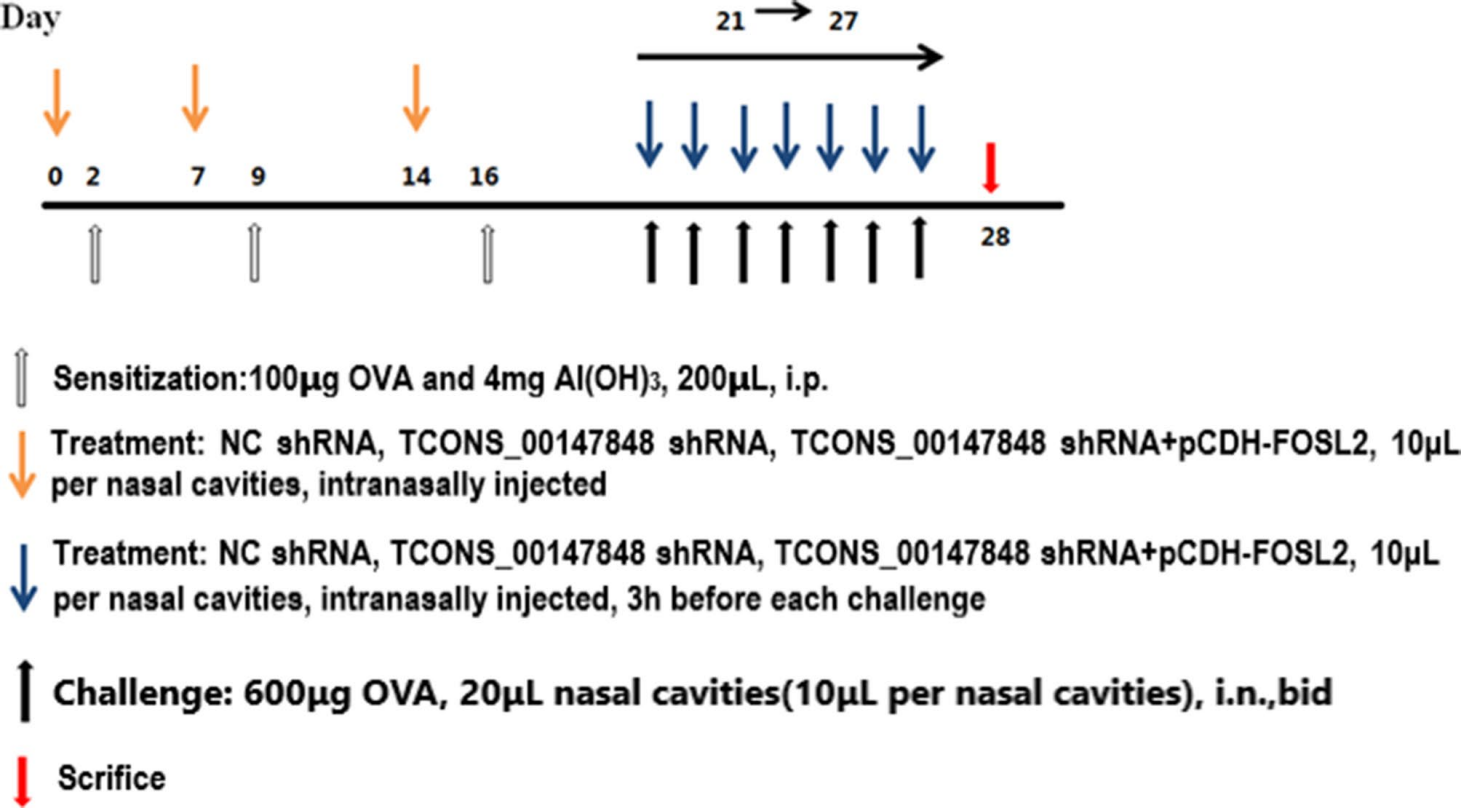
Schematic diagram of OVA-induced allergic rhinitis model.

All animal protocols were approved by the Animal Experimental Ethics Committee of Inner Mongolia People’s Hospital. SPF BALB / C healthy mice of 6–8 weeks purchased from Shanghai SLAC Laboratory Animal Co., Ltd. (Shanghai, China) were randomly divided into Control group, AR group, AR + NC shRNA group, AR + TCONS_00147848 shRNA group and AR + TCONS_00147848 shRNA + FOSL2 OE group, with 8 mice in each group. Mice in AR group, AR + NC shRNA group, AR + TCONS_00147848 shRNA group and AR + TCONS_00147848 shRNA + FOSL2 OE group were intraperitoneally injected with 200 μl ovalbumin (OVA) / aluminum hydroxide (Al (OH) 3) mixture (with 100 μg OVA and 4 mg Al (OH)_3_) on D2, D9 and D16. On D21-27, 20 μl OVA nasal drops (with 600 μg OVA) were injected into bilateral nasal cavities of mice (10 μl each side of nasal cavity, twice a day). Mice in the control group were injected intraperitoneally and intranasally with the same dose of normal saline. Mice in the AR + NC shRNA group, AR + TCONS_00147848 shRNA group and AR + TCONS_00147848 shRNA + FOSL2 OE group were intranasal administered with NC shRNA, TCONS_00147848 shRNA and TCONS_00147848 shRNA + pCDH-FOSL2, respectively, on D0, D7 and D14 (20 μl each time and 10 μl in each side of nasal cavity), and 20 μ l of NC shRNA, TCONS_00147848 shRNA and TCONS_00147848 shRNA + pCDH-FOSL2 were given, respectively, within 3 h before each challenge from D21 to D27. OVA-induced AR mice model and administration of TCONS_00147848 shRNA and pCDH-FOSL2 schedule are shown in Fig. 1.

### Model evaluation

The success criteria of AR mice model: evaluated by scoring method, observed for 30 min after the last OVA challenge, recorded the number of sneezes, the degree of nasal itching and the amount of nasal secretion, and the total score > 5 points indicates a successful model^11^.

### Hematoxylin–Eosin (HE) staining

Mice in each group were anesthetized and sacrificed within 24 h after the last stimulation, and nasal mucosal tissues were collected. The collected tissue were fixed with 4% formalin in PBS, and then embedded in paraffin. The embedded tissue was sectioned into 5 μm, which underwent Hematoxylin–Eosin (HE) staining according to the method described previously then^12^. Samples on the section were analyzed under a light microscopy.

### Terminal deoxynucleotidyl transferase dUTP nick end labeling (TUNEL) experiment

Tissue apoptosis was detected with the TUNEL assay kit (Sangon Biotech Co. Ltd) as recommended by the manufacturer. The nasal mucosal tissues were fixed with 4% paraformaldehyde in PBS at 37 °C for 1 h, and permeabilized with 0.1% Triton X-100 in 0.1% sodium citrate for 2 min. After washing with PBS, TUNEL reaction mixture was incubated at 37 °C for 1 h, and the stained cells were observed with fluorescence microscopy (Eclipse TE300, Nikon, Japan).

### Culture, purification and identification of nasal mucosal epithelial cells of mice

Normal mice were sacrificed after cervical dislocation. Nasal mucosa tissues were isolated under sterile conditions and washed with phosphate buffer (PBS), and then were cut into pieces and digested with 0.1% collagenase I (incubated at 37 °C for 30 min). After that, the same amount of 0.25% trypsin was added, and the cells were collected by centrifugation. DMEM / F12 culture medium with 10% fetal bovine serum (FBS) was washed 3 times, and preattached at 37 °C for 30–60 min to remove the contamination of fibroblasts. The cell density was adjusted to 10^6^ cells/mL with DMEM/F12 medium, and the cells were inoculated in 6-well cell culture plates. Then, the culture plates cultured in a constant temperature incubator at 37 °C with 5% CO_2_. The solution was changed every 2–3 days, and cells were subcultured when they grew into the monolayer. All the cells used in the experiment grew steadily between the second and third generations. The cell morphology, proliferation and growth were observed under inverted microscope.

Nasal mucosa epithelial cells of mice were identified by immunofluorescence method. The cover glass slides containing primary nasal mucosa epithelial cells were cleaned with PBS and fixed with 4% paraformaldehyde solution for 30 min, and permeabilized with 0.1% Triton X-100 at room temperature for 20 min. The cells were incubated with CK18 primary antibody (1:100) overnight at 4 °C, then cleaned with PBS and incubated at room temperature with FITC-labeled secondary antibody (1:100) for 2 h. After that, the nuclei were stained with DAPI and cleaned with PBS. Leica TCS SPE confocal microscope (Heerbrugg, Switzerland) was used for visualization.

### Construction and grouping of cell models

The cells were cultured in DMEM / F12 culture medium with 10% FBS to a fusion degree of about 70%. Then, NC shRNA, TCONS_00147848 shRNA and pCDH-FOSL2 were transfected into the cells. When stably transfected cells were obtained, they were replaced with serum-free and antibiotic-free medium with TNF-α (10 ng / ml). The control group was cultured as the same steps. The cultured supernatant and cell specimens were collected 24 h later for detection. HNEPC were divided into control group, TNF-α group, TNF-α + NC shRNA group, TNF-α + TCONS_00147848 shRNA group and TNF-α + TCONS_00147848 shRNA + FOSL2 OE group.

### Cell counting kit-8 (CCK-8) to detect cell proliferation

All cells were inoculated in a 96-well plates at 4 × 10^3^ cells/well and cultured in full RPMI-1640 medium. CCK-8 was added at 24 h, 48 h, and 72 h, respectively. The absorbance at 450 nm was measured with a microplate reader (Thermo Fisher Scientific).

### Transwell to detect cell migration

The upper chamber of Transwell chamber (Fermentas, USA) was coated with uniformly diluted Matrigel (BD Biosciences, USA), and the upper chamber Matrigel was hydrated in serum-free medium. Each group was provided with 3 parallel holes, with adding 100 μl of 1 × 10^6^/mL cell suspension to the upper chamber, and 600 μl culture medium with 10% FBS to the lower chamber, which were incubated at 37℃ with 5% CO_2_ for 24 h. Transwell chamber was removed, and the matrix glue and cells in the upper chamber were wiped with a cotton swab, fixed with methanol for 30 min, and stained with Giemsa for 30 min. The cells passing through the filtration membrane were counted under a microscopy (taking the average number of 8 fields as the mean).

### Apoptosis analysis by Annexin V/PI

Cells were inoculated on plates for 12 h, treated with 0.25% trypsin for 24 h, and then washed with PBS for 3 times, which were resuspended with 300 μl binding buffer, then mixed gently with 5 μl Annexin V-FITC (CA, USA), and finally 10 μl PI staining solution added. After that, the cells were placed in the dark at room temperature for 15 min. The apoptosis rate was detected by flow cytometry (BD Biosciences, USA) and analyzed by BD ACCURI C6 software.

### Enzyme-linked immunosorbent assay (ELISA)

The levels of IgE, IL-4, IL-5, IL-10, IFN-γ, TNF-α and IL-9 in the serum were determined by ELISA kits (Abcam, USA) according to the manufacturer’s instructions.

### Quantitative real-time PCR (qRT-PCR)

Total RNA was extracted through TRIzol® Reagent (CA, USA). cDNA was synthesized from 0.5 μg mRNA with a cDNA synthesis kit (Applied Biosystems, Japan). qRT-PCR was performed with an ABI Prism 7500 instrument (Applied Biosystems, Japan). The incubation was initiated at 37 °C for 15 min, followed by 95 °C for 5 min, 94 °C for 30 s, 56 °C for 30 s, for 35 cycles. Finally, it was extended at 72 °C for 7 min. The relative mRNA level was quantified by 2^−△△Ct^. GAPDH served as internal control. The primers used in the present study are as follow: TCONS_00147848 forward: 5’-TGGTGCGGGGCTGGAGAATAAAGAG -3’, reverse: 5’-TACCACGCCCCGACCTCTTATTTCT-3’; GAPDH forward: 5’-CCTCAAGATTGTCAGCAAT-3’, reverse: 5’-CCATCCACAGTCTTCTGAGT-3’.

### RNA-binding protein immunoprecipitation (RIP) assay

RIP experiment was conducted with Magna RNA-binding protein immunoprecipitation kit (Millipore, Billerica, MA, USA) according to the manufacturer's instructions. Generally, 80–90% confluent cells were collected and lysed with RIP lysis buffer, and the cell concentration was adjusted to 2.0 × 10^7^ cells/L. Then, 100 μL cell lysate was incubated by RIP buffer with magnetic beads conjugated with anti-FOSL2 (ab222699, Abcam) or negative control normal rabbit IgG (Cat. #PP64B, Millipore). The samples were incubated with proteinase K to digest the protein, then the immunoprecipitated RNA was isolated and analyzed by qRT-PCR. The primer sequence of FOSL2 was described as before.

### RNA pull-down assay

Nuclear extracts were isolated from nasal mucosa cells of mice. Firstly, a biotin-RNA pull-down assay was performed with 1.5 mg biotinylated RNA antisense. The mixture was then incubated with yeast tRNA (Sigma), which was pre-blocked by streptavidin magnetic beads (Invitrogen). After three washings with EMSA buffer, the beads were collected and the RNA and protein complexes eluted. Finally, the RNA protein binding mixtures were identified by Western blot assay.

### Co-immunoprecipitation (CoIP) assay

Nasal mucosa cells of mice were transfected with FOSL2 and STAT3 plasmids, seperately. The pcDNA3.1 plasmid was transfected as a control. After transfection of 48 h, cells were washed by PBS twice and lysed in lysis buffer (20 mM/L Tris–HCl, 1% NP40, 150 mmol/L NaCl, 10% Glycerol and protease inhibitor cocktail) for 30 min with gentle shaking, which were clarified by centrifugation at 15,000 g at 4 °C for 10 min, and the supernatant was precleared with Protein G-agarose beads. The antibody of target protein was added for immunoprecipitation. The precipitation was analyzed by SDS-PAGE and Western blot.

### Western blot assay

Total protein was extracted with RIPA lysate. The quantitative analysis was performed with BCA protein quantitative kit. After denaturation, the proteins were separated by 10% SDS-PAGE electrophoresis and transferred to PVDF membrane (Washington, NY). At room temperature, the cell membrane was blocked with a TBS Tween‐20 buffer containing 5% bovine serum albumin (BSA) for 1 h before adding the monoclonal antibodies, which were purchased from Cell Signaling Technology and used at manufacturer-recommended dilutions. After washing, the blot was incubated with HRP-conjugated secondary antibody (Santa Cruz, USA) at room temperature for 1 h. Then, protein bands were developed with ECL reagents, and images were acquired with the Bio-Rad Imaging system.

### Statistical analysis

All statistical analyses were performed using SPSS 23.0. Data were expressed as mean ± SD. Results were analyzed by one-way ANOVA with S-N-K multiple comparison test. *p* < 0.05 was considered as significant difference.

### Statement

The study was carried out in compliance with the ARRIVE guidelines. All methods were in accordance with the Guide for the Care and Use of Laboratory Animals issued by the Institute of Laboratory Animal Resources of the Life Science Committee of the National Research Council.

## Results

### Effects of TCONS_00147848 and FOSL2 on anaphylaxis symptom score, TCONS_00147848 expression nasal mucosa histopathology and apoptosis in mice

**Figure 2:**
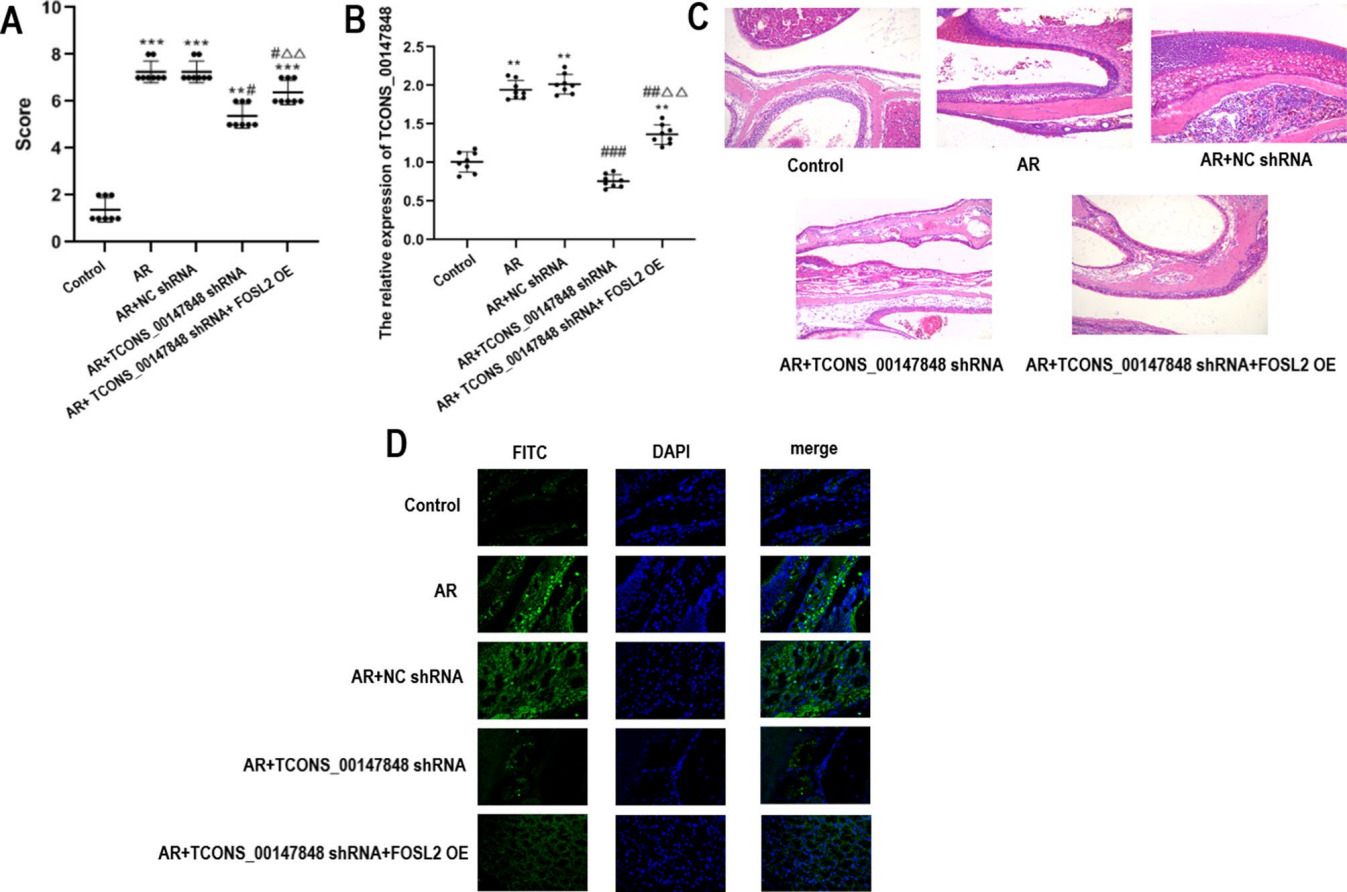
TCONS_00147848 and FOSL2 regulated allergic reaction symptom score, TCONS_00147848 expression, nasal mucosa histopathology, and apoptosis in mice. (**A**) Anaphylaxis symptom score of mice in each group. (**B**) Expression of TCONS_00147848 detected by RT-PCR. (**C**) Pathological changes in nasal mucosa of mice detected by HE staining. (**D**) Apoptosis in nasal mucosa of mice detected by TUNEL staining. ** *P* < 0.01 vs. Control group, *** *P* < 0.001 vs. Control group, # *P* < 0.05 vs. AR group, ## *P* < 0.01 vs. AR group, ### *P* < 0.001 vs. AR group, △△*P* < 0.01 vs. AR + TCONS_00147848 shRNA group (Software version: SPSS 23.0; URL: https://www.ibm.com/legal/copytrade.shtml).

The symptoms of anaphylaxis were scored by recording the sneezing times, nasal itching and nasal secretions of mice, as shown in Fig. 2A. Compared with the control group, the scores of AR group, AR + NC shRNA group, AR + TCONS_00147848 shRNA group and AR + TCONS_00147848 shRNA + FOSL2 OE group significantly increased (*P* < 0.01). Compared with the AR group, the score of AR + NC shRNA group had no significant change, and the scores of AR + TCONS_00147848 shRNA group and TCONS_00147848 shRNA + FOSL2 OE group were significantly lower (*P* < 0.05). However, compared with the AR + TCONS_00147848 shRNA group, the score of AR + TCONS_00147848 shRNA + FOSL2 OE group significantly increased (*P* < 0.05). The relative expression of TCONS_00147848 is shown in Fig. 2B. Compared with the control group, the expression of TCONS_00147848 in AR group, AR + NC shRNA group and AR + TCONS_00147848 shRNA + FOSL2 OE group was significantly up-regulated (*P* < 0.05), without significant difference in AR + TCONS_00147848 shRNA group. The pathological results under 100X-time visual field are shown in Fig. 2C. Compared with the control group, nasal secretions of AR group significantly increased, with obvious nasal mucosal tissue hyperplasia, the degree of inflammation in the nasal mucosa significantly increased, the epithelial cells of the nasal mucosa were destroyed and shed, and the submucosal eosinophils were infiltrated. Compared with the AR group, nasal mucosal inflammation significantly decreased in the AR + TCONS_00147848 shRNA group, but FOSL2 overexpression affected the effect of TCONS_00147848 shRNA. Apoptosis of tissue cells is shown in Fig. 2D. Compared with the control group, apoptotic cells significantly increased in AR group, AR + NC shRNA group, AR + TCONS_00147848 shRNA group and AR + TCONS_00147848 shRNA + FOSL2 OE group. The number of apoptotic cells in AR group and AR + NC shRNA group was significantly higher than that in AR + TCONS_00147848 shRNA group. The number of apoptotic cells in AR + TCONS_00147848 shRNA + FOSL2 OE group was significantly lower than that in AR + TCONS_00147848 shRNA group. These results suggest that TCONS_00147848 knockdown can inhibit the apoptosis of nasal mucosa cells, while FOSL2 overexpression can interfere with this result.

### Effects of TCONS_00147848 and FOSL2 on expression of inflammatory factors in mice

**Figure 3:**
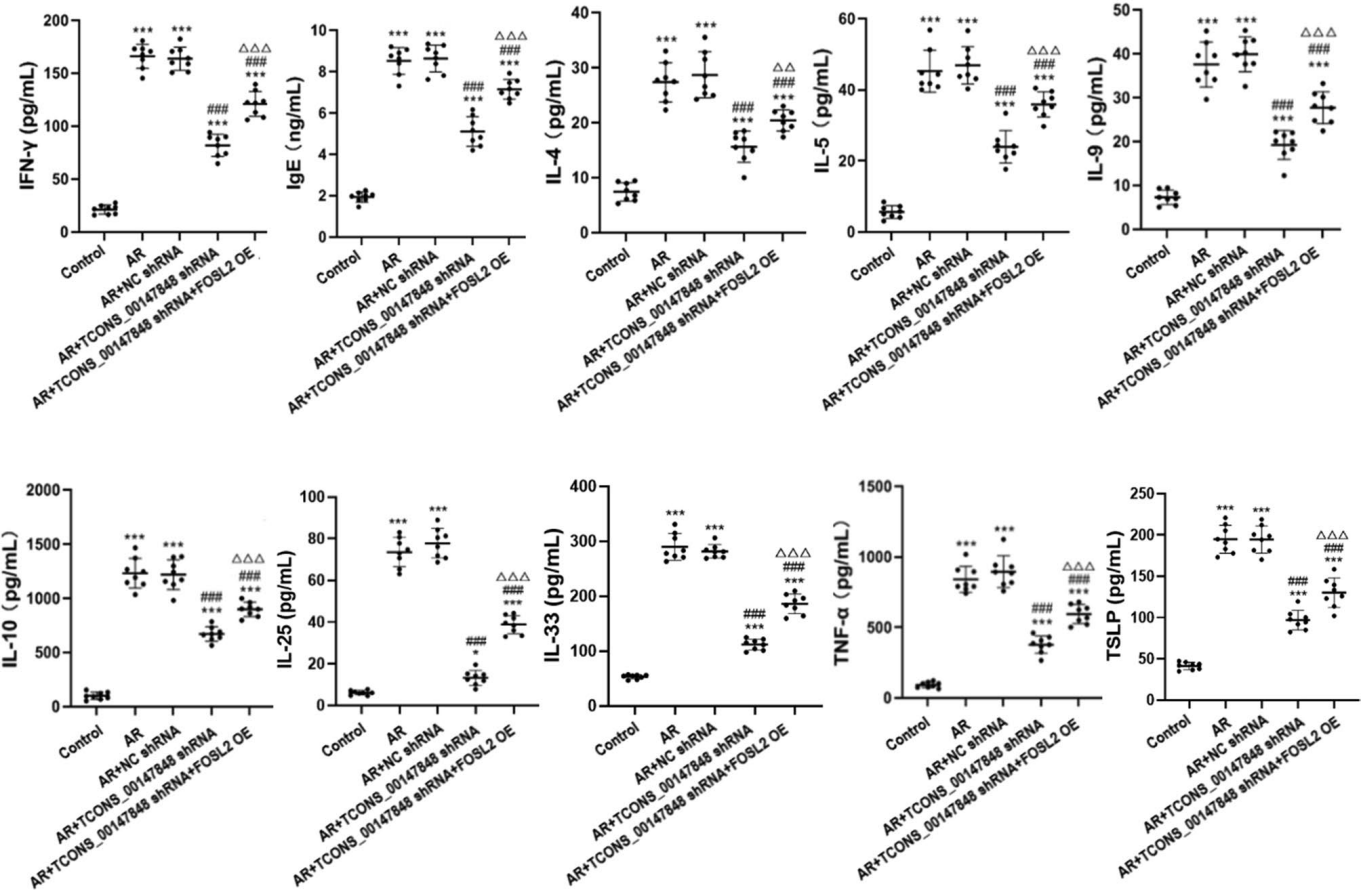
TCONS_00147848 and FOSL2 regulated expressions of IgE, IL-4, IL-5, IL-9, IL-10, IL-25, IL-33, TSLP, IFN-γ and TNF-α detected by ELISA in mice. * *P* < 0.05 vs. Control group, *** *P* < 0.001 vs. Control group, ### *P* < 0.001 vs. AR group, △△*P* < 0.01 vs. AR + TCONS_00147848 shRNA group, △△△*P* < 0.001 vs. AR + TCONS_00147848 shRNA group (Software version: SPSS 23.0; URL: https://www.ibm.com/legal/copytrade.shtml).

ELISA results are shown in Fig. 3. Compared with the control group, the expression levels of IgE, IL-4, IL-5, IL-9, IL-10, IL-25, IL-33, IFN-γ, TNF-α and TSLP were significantly up-regulated in AR group, AR + NC shRNA group, AR + TCONS_00147848 shRNA group and AR + TCONS_00147848 shRNA + FOSL2 OE group. Compared with the AR group, the expression levels of IgE, IL-4, IL-5, IL-9, IL-10, IL-25, IL-33, IFN-γ, TNF-α and TSLP in AR + TCONS_00147848 shRNA group and AR + TCONS_00147848 shRNA + FOSL2 OE group significantly decreased (*P* < 0.05). However, compared with the AR + TCONS_00147848 shRNA group, the expression levels of IgE, IL-4, IL-5, IL-9, IL-10, IL-25, IL-33, IFN-γ, TNF-α and TSLP in AR + TCONS_00147848 shRNA + FOSL2 OE group significantly increased (*P* < 0.05).

### Effects of TCONS_00147848 and FOSL2 on expression of FOSL2, JAK-2, STAT3, P-STAT3, BAX, and BCL-2 in mice

**Figure 4:**
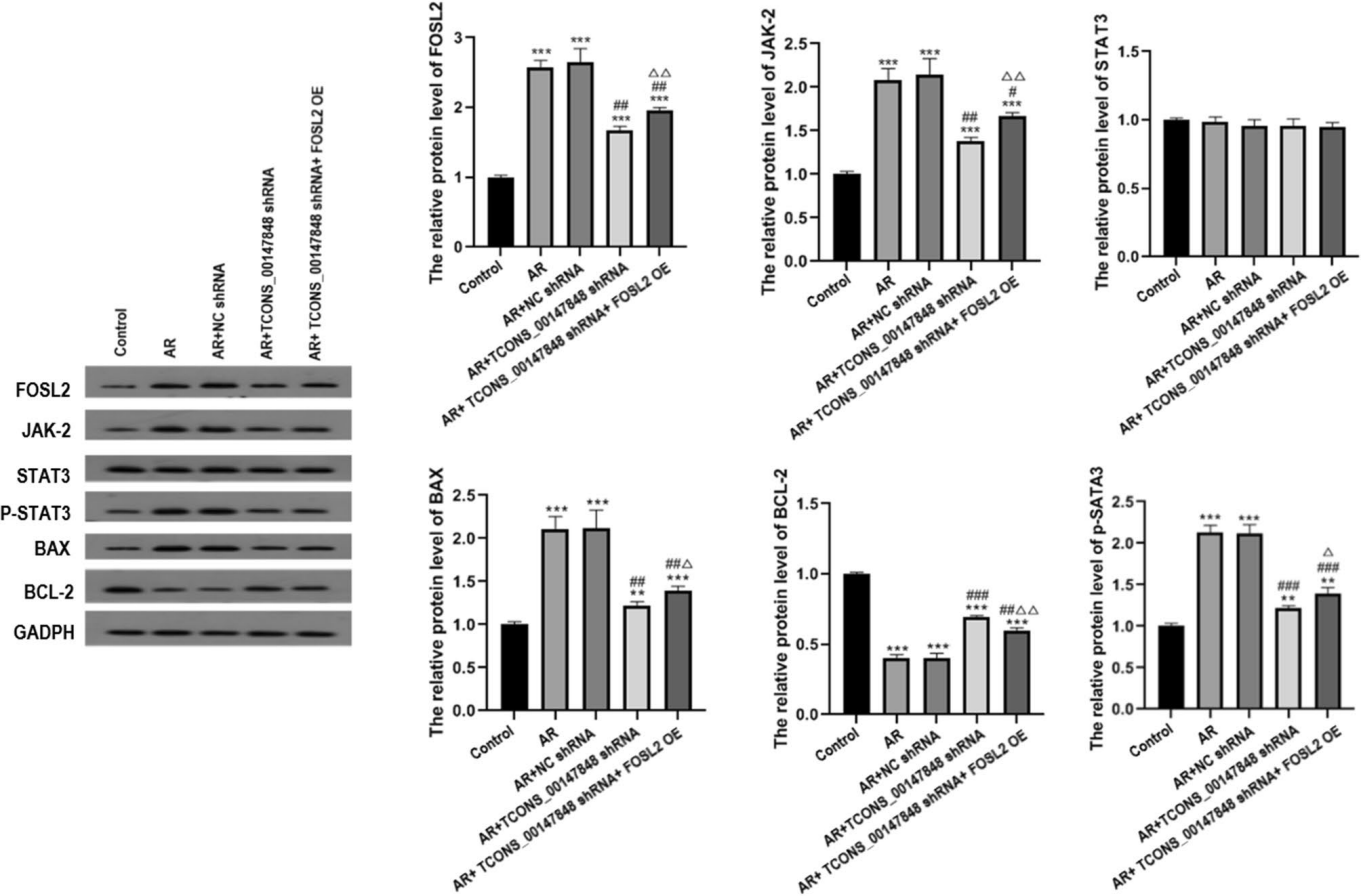
TCONS_00147848 and FOSL2 regulated expressions of FOSL2, JNK-2, STAT3, P-STAT3, BAX, and BCL-2 in mice detected by Western blot. ** *P* < 0.01 vs. Control group, # *P* < 0.05 vs. AR group, ## *P* < 0.01 vs. AR group, ### *P* < 0.001 vs. AR group, △*P* < 0.05 vs. AR + TCONS_00147848 shRNA group, △△*P* < 0.01 vs. AR + TCONS_00147848 shRNA group (Software version: SPSS 23.0; URL: https://www.ibm.com/legal/copytrade.shtml).

Compared with the control group, the expressions of FOSL2, JAK-2, P-STAT3, and BAX were significantly up-regulated in AR group, AR + NC shRNA group, AR + TCONS_00147848 shRNA group and AR + TCONS_00147848 shRNA + FOSL2 OE group (*P* < 0.05). Compared with the AR group, the expressions of FOSL2, JAK-2, P-STAT3, and BAX significantly decreased in AR + TCONS_00147848 shRNA group and AR + TCONS_00147848 shRNA + FOSL2 OE group (*P* < 0.05). Compared with the AR + TCONS_00147848 shRNA group, the expressions of FOSL2 , JAK-2, P-STAT3, and BAX significantly increased in AR + TCONS_00147848 shRNA + FOSL2 OE group (*P* < 0.05). However, BCL-2 expression trend was opposite to that of FOSL2, JAK-2, STAT3, P-STAT3 and BAX, without significant difference in STAT3 expression among all groups (Fig. 4).

### Morphological characteristics and identification of primary nasal mucosa epithelial cells of mice

**Figure 5:**
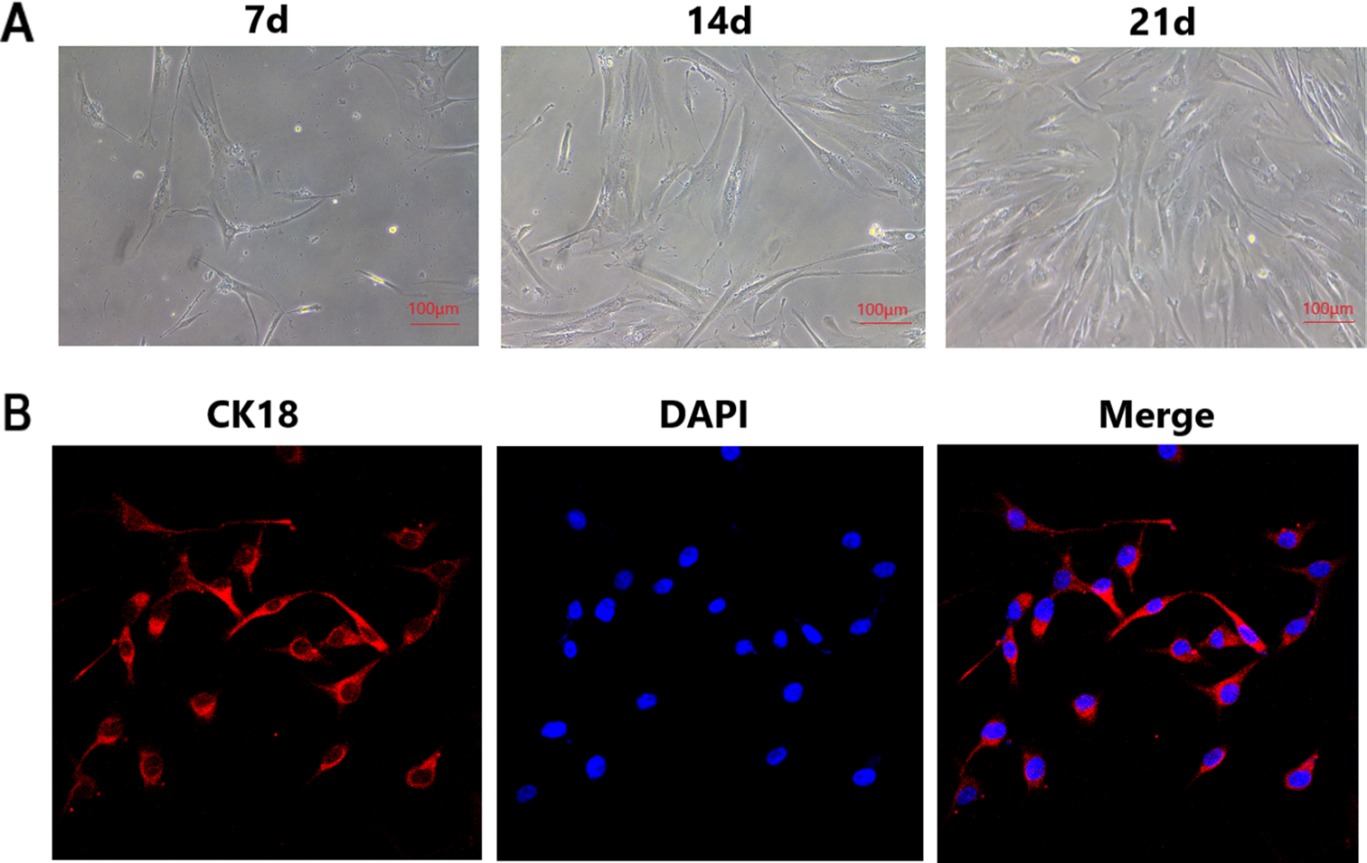
Morphological characteristics and immunocytochemical identification of nasal mucosa epithelial cells. (**A**) Morphological observation of nasal mucosa epithelial cells of mice under inverted microscope. (**B**) Identification of nasal mucosa epithelial cells of mice by immunostaining for CK18 under fluorescence microscope.

On the 7th day of culture, nasal mucosa epithelial cells could be seen. On the 14th day of culture, the cells could grow up to about 50%. On the 21st day of culture, the cells could fill the culture plates (Fig. 5A). The nasal mucosa epithelial cells have clear margin, large quantity and good activity. CK18 was used to identify cultured nasal mucosa epithelial cells of mice, and the expression of CK18 in cytoplasm showed red fluorescence under fluorescence microscope, which confirmed that the cells were epithelial-derived cells. In the same field of vision, DAPI staining of the nuclei showed that the nuclei and cytoplasm with positive expression of CK18 were essentially coincident, indicating that the purity of cultured nasal mucosa epithelial cells was high (Fig. 5B).

### Effects of TCONS_00147848 and FOSL2 on apoptosis, proliferation and migration of nasal mucosa cells of mice treated with TNF—α

**Figure 6:**
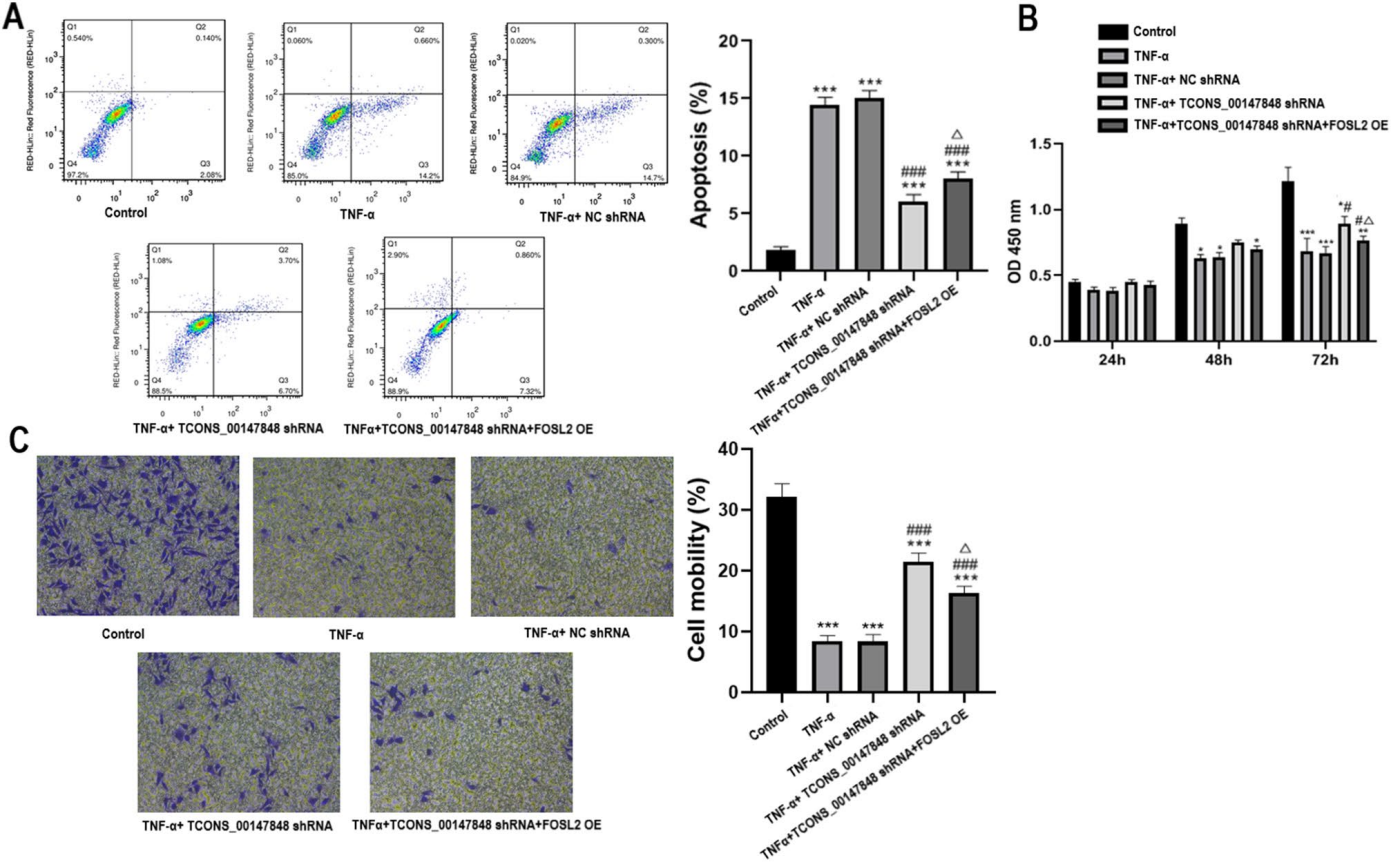
TCONS_00147848 and FOSL2 regulated apoptosis, proliferation and migration of nasal mucosa cells of mice treated with TNF- α. (**A**) Apoptosis of nasal mucosa cells of mice detected by Annexin V/PI (Software version: BD ACCURI C6 software; URL: https://www.AccuriCytometers.com). (**B**) Proliferation of nasal mucosa cells of mice detected by CCK-8. (**C**) Migration of nasal mucosa cells of mice detected by transwell. *P < 0.05 vs. Control group, **P < 0.01 vs. Control group, *** *P* < 0.001 vs. Control group, # *P* < 0.05 vs. TNF-α group, ### *P* < 0.001 vs. TNF-α group, △*P* < 0.05 vs. TNF-α + TCONS_00147848 shRNA group (Software version: SPSS 23.0; URL: https://www.ibm.com/legal/copytrade.shtml).

The results of apoptosis detection are shown in Fig. 6A. Compared with the control group, the apoptotic cells in TNF-α group, TNF-α + NC shRNA group, TNF-α + TCONS_00147848 shRNA group and TNF-α + TCONS_00147848 shRNA + FOSL2 OE group significantly increased (*P* < 0.05). Compared with the TNF-α group, the apoptosis of TNF-α + TCONS_00147848 shRNA group and TNF-α + TCONS_00147848 shRNA + FOSL2 OE group significantly decreased (*P* < 0.05). Compared with the TNF-α + TCONS_00147848 shRNA group, the apoptosis of TNF-α + TCONS_00147848 shRNA + FOSL2 OE group significantly increased (*P* < 0.05). The results of cell proliferation and migration are shown in Fig. 6B,C. Compared with the control group, the proliferation and migration of cells in TNF-α group, TNF-α + NC shRNA group, TNF-α + TCONS_00147848 shRNA group and TNF-α + TCONS_00147848 shRNA + FOSL2 OE group significantly decreased (*P* < 0.05). Compared with the TNF-α group, the proliferation and migration of TNF-α + TCONS_00147848 shRNA group and TNF-α + TCONS_00147848 shRNA + FOSL2 OE group significantly increased (*P* < 0.05). Compared with the TNF-α + TCONS_00147848 shRNA group, the proliferation and migration of TNF-α + TCONS_00147848 shRNA + FOSL2 OE group significantly decreased (*P* < 0.05).

### Effects of TCONS_00147848 and FOSL2 on expression of inflammatory factors and TCONS_00147848 in nasal mucosa cells of mice treated with TNF-α

**Figure 7:**
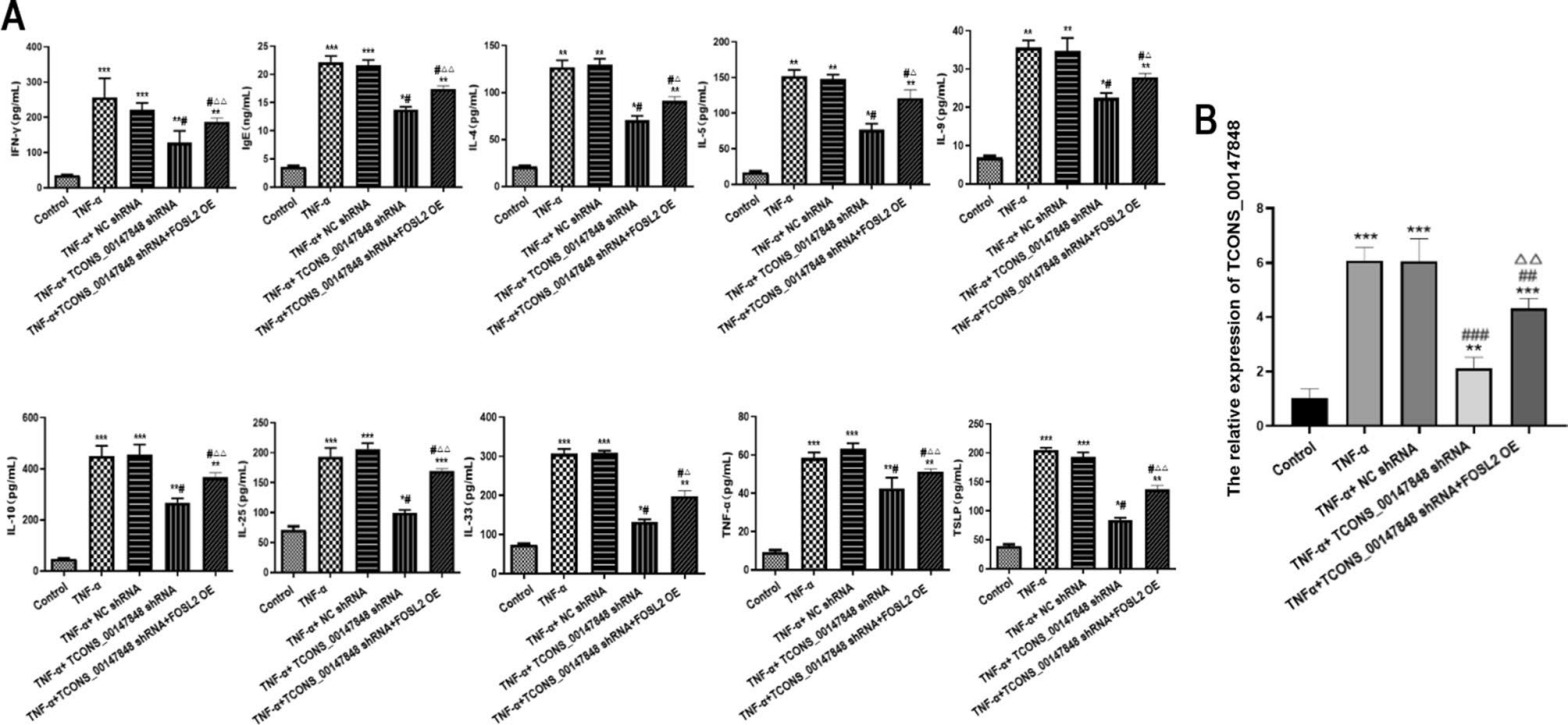
TCONS_00147848 and FOSL2 regulated expression of inflammatory factors and TCONS_00147848 in nasal mucosa cells of mice treated with TNF-α. (**A**) Expression of IgE, IL-4, IL-5, IL-9, IL-10, IL-25, IL-33, TSLP, IFN-γ and TNF-α detected by ELISA. (**B**) Expression of TCONS_00147848 detected by RT-PCR. * *P* < 0.05 vs. Control group, ** *P* < 0.01 vs. Control group, *** *P* < 0.001 vs. Control group, # *P* < 0.05 vs. AR group, △*P* < 0.05 vs. TNF-α + TCONS_00147848 shRNA group, △△*P* < 0.01 vs. TNF-α + TCONS_00147848 shRNA group (Software version: SPSS 23.0; URL: https://www.ibm.com/legal/copytrade.shtml).

Compared with the control group, the expression levels of IgE, IL-4, IL-5, IL-9, IL-10, IL-25, IL-33 IFN-γ, TNF-α, TSLP and TCONS_00147848 were significantly up-regulated in TNF-α group, TNF-α + NC shRNA group, TNF-α + TCONS_00147848 shRNA group and TNF-α + TCONS_00147848 shRNA + FOSL2 group (*P* < 0.05). Compared with the TNF-α group, the expression levels of IgE, IL-4, IL-5, IL-9, IL-10, IL-25, IL-33 IFN-γ, TNF-α, TSLP and TCONS_00147848 in TNF-α + TCONS_00147848 shRNA group and TNF-α + TCONS_00147848 shRNA + FOSL2 OE group significantly decreased (*P* < 0.05). However, compared with the TNF-α + TCONS_00147848 shRNA group, the expression levels of IgE, IL-4, IL-5, IL-9, IL-10, IL-25, IL-33 IFN-γ, TNF-α, TSLP and TCONS_00147848 in TNF-α + TCONS_00147848 shRNA + FOSL2 OE group significantly increased (*P* < 0.05) (Fig. 7A,B).

### Effects of TCONS_00147848 and FOSL2 on expression of FOSL2, JAK-2, STAT3, P-STAT3, BAX, and BCL-2 in nasal mucosa cells of mice treated with TNF-α

**Figure 8:**
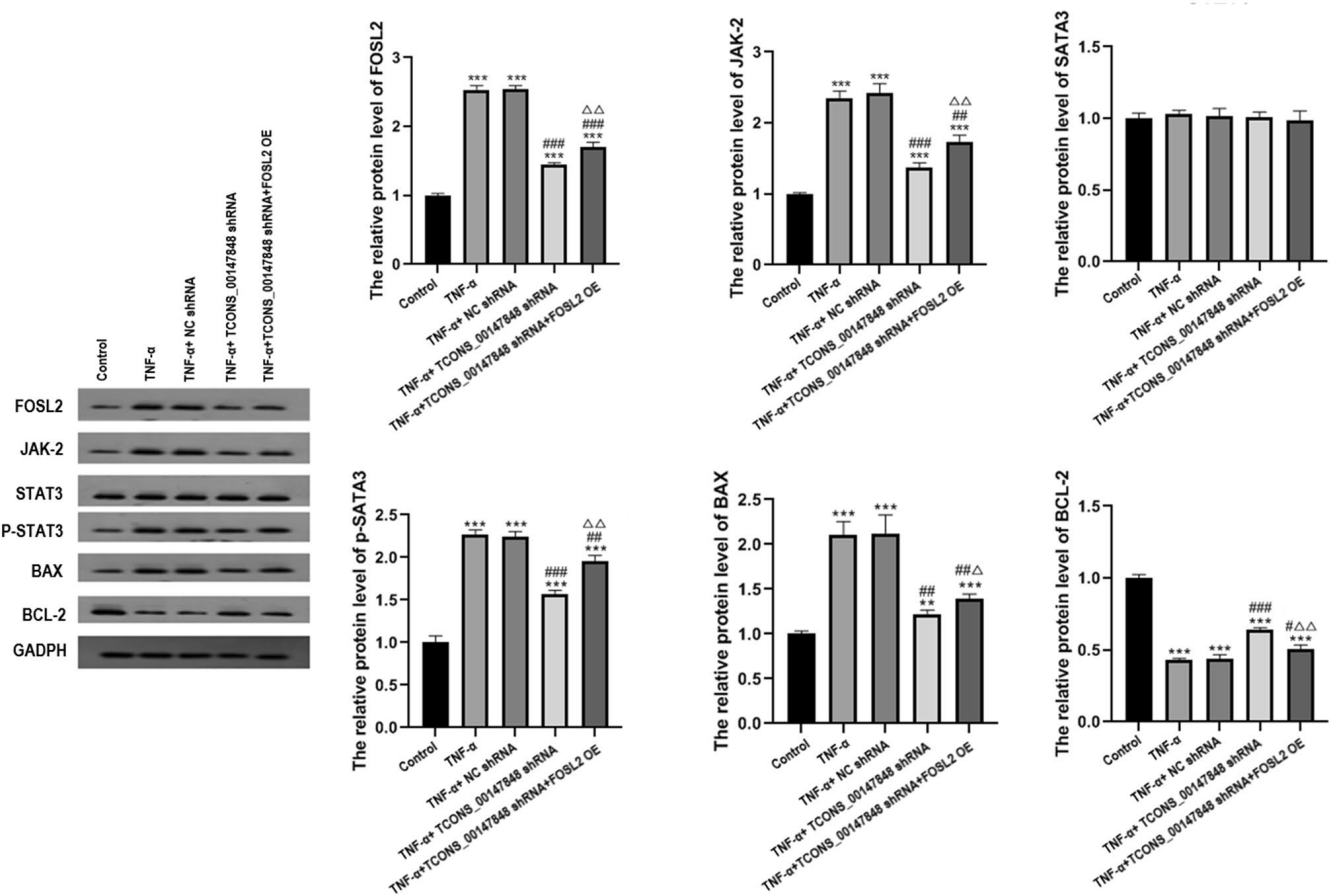
TCONS_00147848 and FOSL2 regulated expressions of FOSL2, JNK-2, STAT3, P-STAT3, BAX, and BCL-2 in nasal mucosa cells of mice treated with TNF-α detected by Western blot. ** *P* < 0.01 vs. Control group, *** *P* < 0.001 vs. Control group, # *P* < 0.05 vs. AR group, ## *P* < 0.01 vs. AR group, ### *P* < 0.001 vs. AR group, △*P* < 0.05 vs. TNF-α + TCONS_00147848 shRNA group, △△*P* < 0.01 vs. TNF-α + TCONS_00147848 shRNA group (Software version: SPSS 23.0; URL: https://www.ibm.com/legal/copytrade.shtml).

Compared with the control group, the expressions of FOSL2, JAK-2, P-STAT3, and BAX were significantly up-regulated in TNF-α group, TNF-α + NC shRNA group, TCONS_00147848 shRNA group and TNF-α + TCONS_00147848 shRNA + FOSL2 OE group (*P* < 0.05). Compared with the TNF-α group, the expressions of FOSL2, JAK-2, P-STAT3, and BAX in TNF-α + TCONS_00147848 shRNA group and TNF-α + TCONS_00147848 shRNA + FOSL2 OE group significantly decreased (*P* < 0.05). However, BCL-2 expression trend was opposite to that of FOSL2, JAK-2, P-STAT3, and BAX, without significant difference in STAT3 expression among all groups (Fig. 8).

### FOSL2 targeted by TCONS_00147848 in nasal mucosa cells of mice

**Figure 9:**
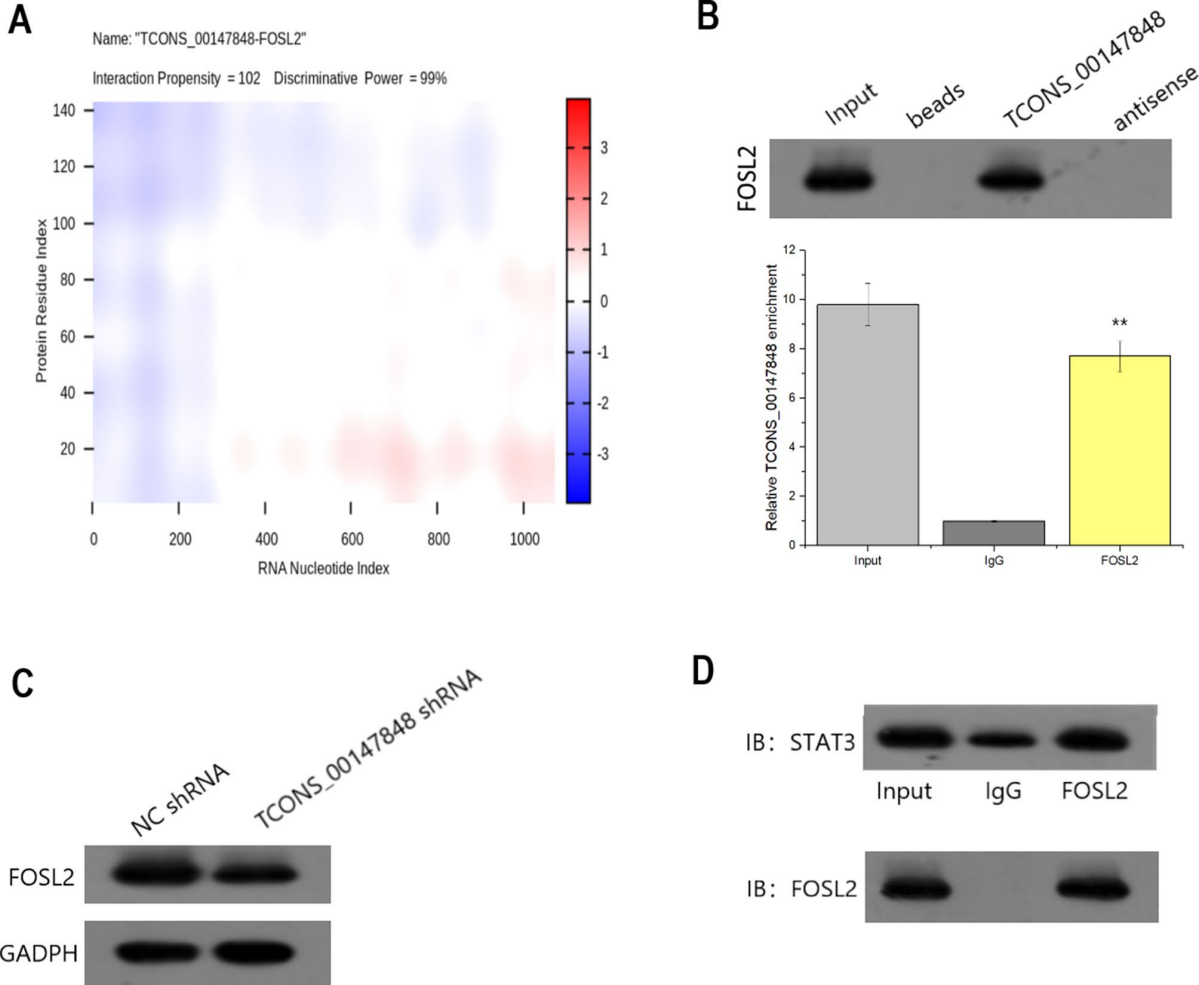
TCONS_00147848 could target FOSL2 in nasal mucosa cells of mice. (**A**) Prediction of the interaction between TCONS_00147848 and FOSL2. A high score obtained for the interaction between TCONS_00147848 and FOSL2. Red color represents the area of the interaction. (**B**) RNA pull-down and RIP assays conducted to prove the interaction between TCONS_00147848 and FOSL2. (**C**) Effect of TCONS_00147848 on FOSL2 protein in nasal mucosa cells of mice explained with Western blot. (**D**) Interplay between FOSL2 and STAT3 confirmed by CoIP assay. ** *P* < 0.01 (Software version: SPSS 23.0; URL: https://www.ibm.com/legal/copytrade.shtml).

In this study, the interaction between TCONS_00147848 and protein based on secondary structure, hydrogen bonds, and intermolecular atomic forces were evaluated with the online calculations of catRAPID database (http://service.tartaglialab.com/page/catrapid_group), showing that the binding capacity between TCONS_00147848 and FOSL2 was the strongest (Fig. 9A). Then, we further verified this interaction in nasal mucosa cells of mice by RNA pull down and RIP assays (Fig. 9B). Also, we found that FOSL2 was positively regulated by TCONS_00147848 in nasal mucosa cells of mice, as its protein level decreased under TCONS_00147848 inhibition but increased upon TCONS_00147848 stimulation (Fig. 9C). Furthermore, the interaction of FOSL2 with STAT3 in nasal mucosa cells of mice was also proofed by CoIP assay (Fig. 9D) (The original Western blot images were shown in Supplementary Material). Thus, we speculated that TCON_00147848 requires FOL2 to activate JAK/STAT3 with signaling.

## Discussion

AR is a common disease of otorhinolaryngology, which is a complex polygenic hereditary disease affected by both heredity and environment. lncRNA is a hot spot in current research^13^, which can regulate gene expression through epigenetic, transcriptional and post transcriptional levels. Evidence has shown that lncRNA was abnormally expressed in many complex diseases, such as tumors, neurodegenerative diseases, cardiovascular diseases and so on, some of which can promote or inhibit the occurrence of diseases^14–16^. However, the effect of lncRNA on AR pathology has not been reported so far. In our previously unpublished lncRNA sequencing results, TCONS_00147848 was first found to highly express in the nasal mucosa of AR patients. This study further explored the mechanism of TCONS_00147848 in AR.

AR is a common type I hypersensitivity reactions. When atopic individuals inhaled the allergens, antigen presenting cells (APC) ingested and processed the allergens and presented them to the initial CD4^+^T cells, which differentiated into Th2 cells under the stimulation of IL-4, of which, the secrete cytokines (such as IL-4, IL-5, IL-9, IL-10, etc.) stimulated B-lymphocyte activation, synthesized specific IgE and released it, thereby sensitizing nasal mucosa. Studies have shown that lncRNA plays an important role in innate immunity and adaptive immunity^17^. In this study, we found that the expressions of IgE, IL-4, IL-5, IL-9 and IL-10 in serum of mice in the AR group was significantly higher than that in the control group, while TCONS_00147848 konckdown could decrease their expressions.

JAK / STAT is a cytokine signaling pathway widely involved in cell proliferation, differentiation, apoptosis and immune regulation^18^. JAK2 is a kind of intracytoplasmic non receptor soluble tyrosine protein kinase expressed in all animal tissues and cells. STAT is a kind of cytoplasmic protein as an important substrate of JAK in JAK / STAT signal transduction pathway, which is involved in the regulation of target gene transcription, so as to achieve cytokine receptor-mediated signal transduction and complete the body's acquired immune response^19^. Studies have shown that JAK / STAT signaling pathway is closely related to inflammatory response. Currently, many inflammatory factors, including tumor necrosis factor—α (TNF—α), can induce inflammatory reaction by activating JAK / STAT signaling pathway^20^. Respiratory diseases are closely related to air pollution, of which, NO_2_ plays a major role. Studies have shown that inhalation of NO_2_ can activate JAK / STAT signaling pathway, induce the abnormal expression of JAK, STAT1 and STAT3, and produce a large number of inflammatory factors, including IFN—γ, which participate in airway inflammation^21^. Thus, we found in this study that the expressions of TNF—α and IFN—γ in serum of mice in the AR group was significantly higher than that in the control group. The expressions of JAK-2 and p-STAT3 proteins significantly increased in nasal mucosa of mice, which is consistent with the results of the study by Shi et al.^22^.

As a member of AP-1 family, FOSL2 was found to be closely related to CD1D-restricted INKT cells. Lawson et al. found that specific knockdown of FOSL2 in CD4^+^T cells resulted in spontaneous increase thymus and peripheral INKT cells, but did not affect other T cell lines^23^. Under chronic inflammatory conditions, FOSL2 overexpression can promote the deterioration of the disease by upregulating the expression of ICOs molecules on T cells^24^. In CD4 + T cells, FOSL2 is regulated by IL-2 as a target gene of STAT5, which may also be involved in the regulation of Th7 differentiation^25^. In this study, FOSL2 was the target gene of TCONS_00147848, which has been shown that transcriptional and DNA-binding activity of AP-1 in T cells is associated with increased phosphorylation of STAT1^26^. Liang et al. demonstrated that miR-30e could inhibit the activation of the JSK2/STAT3 signaling pathway by decreasing FOSL2 expression, thereby inhibiting the apoptosis of liver cells and promoting the proliferation of liver cells^6^. However, whether FOSL2 can regulate AR through JAK / STAT signaling pathway has not been reported. And it was found in our study that FOSL2 protein expression in nasal mucosa of the AR group was significantly higher than that of the control group. When the expression of TCONS_00147848 in AR mice was knocked down, the expressions of TNF—α and IFN—γ in serum and the protein expressions of FOSL2, JAK-2, and p-STAT3 in nasal mucosa of AR mice also significantly decreased, which confirmed that inhibition of TCONS_00147848 expression can target regulation of FOSL2 mediated inflammatory response and alleviate allergic symptoms in AR mice. However, the limitation of this study is that it has not proved whether TCONS_00147848 is involved in the pathogenesis of human AR, which is also a question to be verified in our next experiment.

## Conclusion

In conclusion, the expression of TCONS_00147848 in nasal mucosa of AR mice significantly increased, which was involved in the regulation of apoptosis and inflammation of nasal mucosa cells through JAK2/STAT3 signaling pathway. The low expression of TCONS_00147848 is the protective mechanism of AR closely related to the occurrence and development of AR, with a good prospect and potential value for the personalized diagnosis and treatment of AR and the development of targeted drugs.

## Supplementary Information

Supplementary Information.
