# Supplementary material for: Mechanism of TCONS_00147848 regulating apoptosis of nasal mucosa cells and alleviating allergic rhinitis through FOSL2-mediated JAK/STAT3 signaling pathway
Source: Sci Rep. 2021 Aug 6;11:15991. doi: 10.1038/s41598-021-94215-3 (PMC8346477; doi:10.1038/s41598-021-94215-3)

**Mechanism of TCONS_00147848 regulating apoptosis of nasal mucosa cells and alleviating allergic rhinitis through FOSL2-mediated JAK/STAT3 signaling pathway**

Haiyun Huang^1^, Yu Ren^2^, Hongyu Liang^2^, Xiaojia Liu^1^, Jisangmo Nan^2^, Hui Zhao^3^, Xiaoling Liu^1*^

1 ENT Department, Inner Mongolia People’s Hospital, Hohhot Inner Mongolia Autonomous Region, 010017, China

2 Scientific Research Department, Inner Mongolia People’s Hospital, Hohhot Inner Mongolia Autonomous Region, 010017, China

3 Medical Department, Inner Mongolia People’s Hospital, Hohhot, Hohhot Inner Mongolia Autonomous Region, 010017, China

**Corresponding author:**

Xiaoling Liu

ENT Department, Inner Mongolia People’s Hospital, Hohhot Inner Mongolia Autonomous Region, 010017, China

Email: xiaolingliu666@126.com

**Figure 4: As full as possible length gels and blots for Figure 4.**


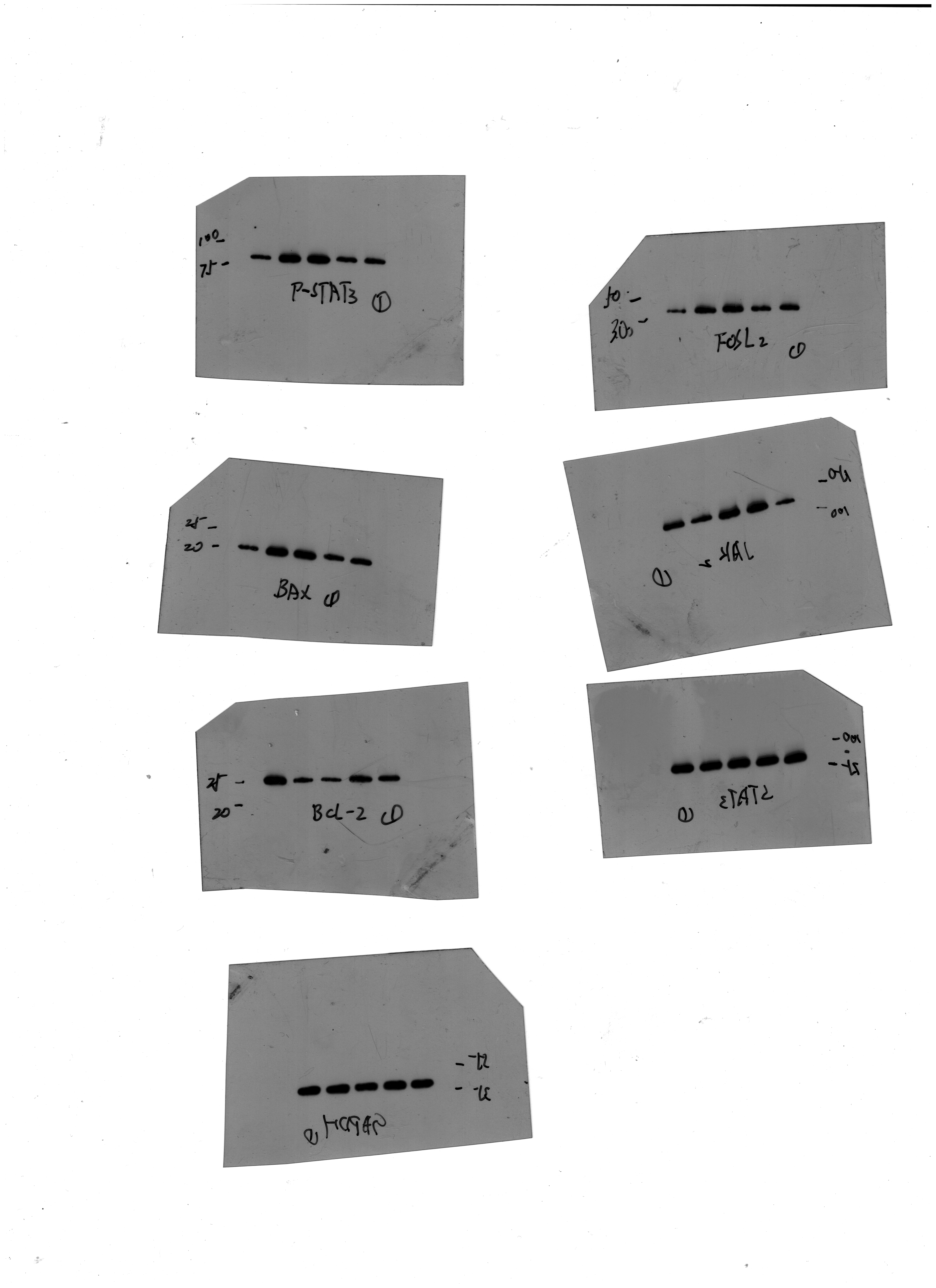


**Figure 8: As full as possible length gels and blots for Figure 8.**

**
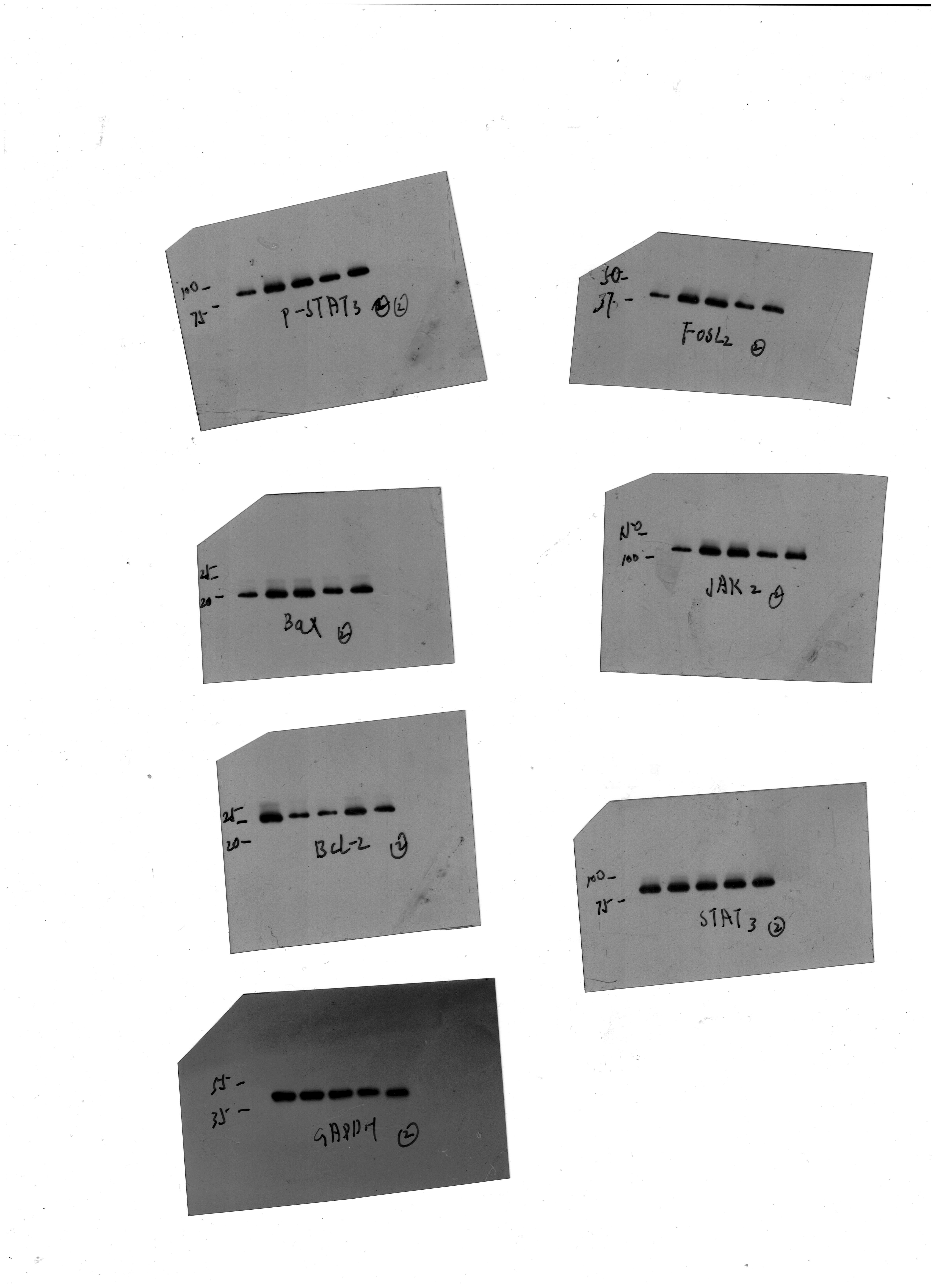
**

**Figure 9: As full as possible length gels and blots for Figure 9.**


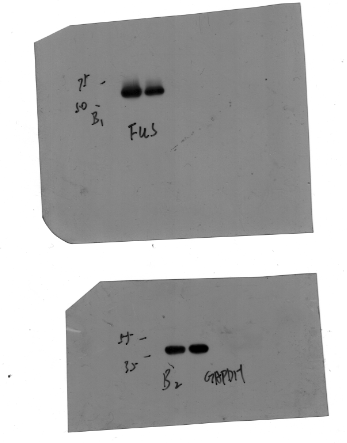

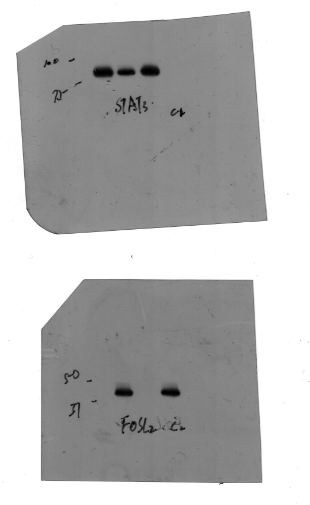


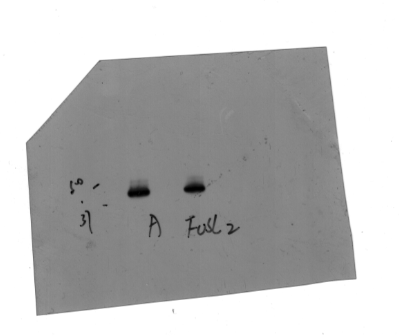

Supplement: Supplementary file 1 — Supplementary Information. [file 41598_2021_94215_MOESM1_ESM.doc]
